# Supplementary material for: Content-rich biological network constructed by mining PubMed abstracts
Source: BMC Bioinformatics. 2004 Oct 8;5:147. doi: 10.1186/1471-2105-5-147 (PMC528731; doi:10.1186/1471-2105-5-147)
Supplement: Additional File 2 — The original results of the above study (non-essential files are deleted to keep the file size under the limit set by BMC bioinformatics). [file 1471-2105-5-147-S2.bz2 › chilibotAdditionalFile2/dip20/56ID8493578E211/html/RB1_E7.html]

 


 **RB1** and **E7** 
  
Found 222 abstracts in PubMed, retrieved 20.  
 

 What does Google say? 
 PDF only 
| .edu only 

---

**Interactive relationship** (e.g. stimulation, inhibition, etc)

**Inhibitory relationship**- The human papillomavirus HPV type 16  **E7**  oncoprotein must inactivate the retinoblastoma  [ **RB1** ]  tumor suppressor Rb pathway to bypass G 1 arrest.  Ref: 12239337 J Virol, 2002
**Neutral relationship**- Human papillomavirus  **E7**  HPV  **E7**  is a viral oncoprotein that plays an important role in cervical carcinogenesis through binding with retinoblastoma  [ **RB1** ]  protein Rb.  Ref: 12526093 Exp Mol Med, 2002
- Expression of the human papillomavirus type 16 E6 and  **E7**  oncogenes initiates and maintains abnormal cell replication, by interacting with the p53 and retinoblastoma  [ **RB1** ]  Rb gene products.  Ref: 12386813 Oncogene, 2002
- Thus, the similarities and differences in comparison to E1A, T antigen, and  **E7**  make pp71 an interesting new tool with which to further dissect the role of the retinoblastoma  [ **RB1** ]  E2F pathway in cellular growth control and carcinogenesis.  Ref: 12612064 Mol Cell Biol, 2003

**Non-interactive relationship** (e.g. studied together, co-existance, homology, etc.)

- Inactivation of both the retinoblastoma  [ **RB1** ]  tumor suppressor and p21 by the human papillomavirus type 16  **E7**  oncoprotein is necessary to inhibit cell cycle arrest in human epithelial cells.  Ref: 12239337 J Virol, 2002
- The high risk HPVs synthesize E6 and  **E7**  oncoproteins to alter the function of cellular regulatory proteins, such as p53 and retinoblastoma  [ **RB1** ]  gene product, respectively.  Ref: 12670501 Biochem Biophys Res Commun, 2003
- Diffuse, strong positivity with p16 in endocervical adenocarcinomas is likely caused by inactivation of the retinoblastoma  [ **RB1** ]  protein by the  **E7**  human papillomavirus oncoprotein, which acts as a p16 transcript repressor.  Ref: 12819388 Int J Gynecol Pathol, 2003
- The oncoproteins of the DNA tumor viruses, adenovirus E1A, simian virus 40 T antigen, and papillomavirus  **E7** , each interact with the retinoblastoma  [ **RB1** ]  family of tumor suppressors, leading to cell cycle stimulation, apoptosis induction, and cellular transformation.  Ref: 12612064 Mol Cell Biol, 2003
- Surprisingly,  **E7**  expression in terminally differentiated myotubes could not reactivate DNA synthesis even though the oncogene bound the retinoblastoma  [ **RB1** ]  protein, reduced its levels, and increased E2F transcriptional activity.  Ref: 12821937 Oncogene, 2003
